# Mechanical forces as information: an integrated approach to plant and animal development

**DOI:** 10.3389/fpls.2014.00265

**Published:** 2014-06-10

**Authors:** Valeria Hernández-Hernández, Denisse Rueda, Lorena Caballero, Elena R. Alvarez-Buylla, Mariana Benítez

**Affiliations:** ^1^Instituto de Ecología, Universidad Nacional Autónoma de MéxicoMexico City, Mexico; ^2^Centro de Ciencias de la Complejidad, Universidad Nacional Autónoma de MéxicoMexico City, Mexico; ^3^Posgrado en Ciencias Biológicas, Universidad Nacional Autónoma de MéxicoMexico City, Mexico; ^4^Posgrado en Ciencias Biomédicas, Universidad Nacional Autónoma de MéxicoMexico City, Mexico; ^5^Departamento de Nanotecnología, Centro de Física Aplicada y Tecnología Avanzada, Universidad Nacional Autónoma de MéxicoMexico City, Mexico; ^6^Departamento de Sistemas Complejos, Instituto de Física, Universidad Nacional Autónoma de MéxicoMexico City, Mexico

**Keywords:** mechanical forces, tensegrity, positional information, multicellular development, stem-cell niches

## Abstract

Mechanical forces such as tension and compression act throughout growth and development of multicellular organisms. These forces not only affect the size and shape of the cells and tissues but are capable of modifying the expression of genes and the localization of molecular components within the cell, in the plasma membrane, and in the plant cell wall. The magnitude and direction of these physical forces change with cellular and tissue properties such as elasticity. Thus, mechanical forces and the mesoscopic fields that emerge from their local action constitute important sources of positional information. Moreover, physical and biochemical processes interact in non-linear ways during tissue and organ growth in plants and animals. In this review we discuss how such mechanical forces are generated, transmitted, and sensed in these two lineages of multicellular organisms to yield long-range positional information. In order to do so we first outline a potentially common basis for studying patterning and mechanosensing that relies on the structural principle of tensegrity, and discuss how tensegral structures might arise in plants and animals. We then provide some examples of morphogenesis in which mechanical forces appear to act as positional information during development, offering a possible explanation for ubiquitous processes, such as the formation of periodic structures. Such examples, we argue, can be interpreted in terms of tensegral phenomena. Finally, we discuss the hypothesis of mechanically isotropic points as a potentially generic mechanism for the localization and maintenance of stem-cell niches in multicellular organisms. This comparative approach aims to help uncovering generic mechanisms of morphogenesis and thus reach a better understanding of the evolution and development of multicellular phenotypes, focusing on the role of physical forces in these processes.

## Broad comparative studies in evolutionary developmental biology—comparing developmental dynamics

Comparative studies have been key to understanding the evolution of phenotypes. Indeed, the growing field of evolutionary developmental biology, often referred to as evo-devo, has integrated and extended different aspects of comparative evolutionary embryology (Gilbert, 2003; Love and Raff, 2003; Raff and Love, 2004). It has also incorporated the comparison of gene and protein sequences, function and expression patterns (Nijhout, 2003; Kramer, 2005; Müller, 2007; Metscher, 2009), largely focusing on relatively well-conserved genes that play a central role in developmental processes (e.g., Carroll, 1995; Lohmann and Weigel, 2002). Comparative studies in evo-devo have also been enriched by the advent of high-throughput technologies, opening avenues in the comparison of genomes, transcriptomes, proteomes, epigenomes and their relation with phenotypic transformation (Cañestro et al., 2007; Artieri and Singh, 2010; Lira-Medeiros et al., 2010; Ormestad et al., 2011). Recently, several authors have pointed at the importance of identifying and comparing developmental modules in order to fully understand how phenotypes arise and evolve. Such modules range from those associated to molecular regulatory networks (Alvarez-Buylla et al., 2009; Kuratani, 2009; De Bruijn et al., 2012; Fischer and Smith, 2012; Niklas and Kutschera, 2012), to dynamical patterning modules that include conserved gene products in conjunction with the physical morphogenetic and patterning processes they mobilize in the context of multicellularity (Newman et al., 2006; Newman and Bhat, 2009; Hernández-Hernández et al., 2012). In order to study the evolution of development and recognize both generic and specific developmental traits in multicellular organisms, it is necessary to compare developmental processes and modules in lineages in which multicellularity has evolved independently, such as in some plants and animals (Meyerowitz, 2002; Newman et al., 2006; Newman and Bhat, 2009).

Mechanical forces have been acknowledged to play a central role in understanding how biological patterns and morphologies emerge and vary along evolution (Thompson, 1942; Green, 1962; Lintilhac, 1974a,b; Beloussov, 2008; Niklas and Spatz, 2012; for a recent review see Mammoto et al., 2013). The conceptual and technical tools now available are enabling a more thorough study of their action, as well as their dynamical feedback with biochemical and genetic developmental processes (Newman and Bhat, 2009; Niklas and Kutschera, 2012; Purnell, 2012; Barrio et al., 2013; Mammoto et al., 2013; Bozorg et al., 2014 and references therein). In this review we aim at comparing the role of mechanical forces (e.g., tension and compression) in the generation of positional information and patterns in plant and animal systems. On the basis of the currently available evidence, we hypothesize that *tensegrity*, a structural principle first put forward by Buckminster Fuller and extensively developed and considered by D. Ingber and collaborators (e.g., Ingber, 2006, 2008; Mammoto et al., 2013), mainly for animal development, may be part of key developmental processes in both lineages. Finally, we present examples of how mechanical forces may be acting in particular plant and animal developmental systems, and discuss the *mechanical isotropy* hypothesis as a potentially generic mechanism acting in the formation and maintenance of stem-cell niches in both plants and animals.

## Tensegrity as a potentially common mechanism for patterning, communication and mechanosensing

Recent studies in plant and animal model systems have contributed to elucidate the role of mechanical forces in biological development (Beysens et al., 2000; Hayashi and Carthew, 2004; Nakayama et al., 2012; Uyttewaal et al., 2012). As organisms grow and develop, cells are subjected to mechanical forces that may affect, for example, the organization of the cytoskeleton, the shape and local properties of the contractile plasma membrane, and cellular communication through membrane channels. In this way, mechanical forces can be translated into biochemical responses that in turn affect the gene regulatory networks associated to cell fate and proliferative behavior (Engler et al., 2006; Ingber, 2008). Moreover, changes in gene activity induced by mechanical forces may determine cellular properties (rigidity, adhesivity, etc.) that feedback to mechanical fields (i.e., a physical quantity that has a value for the total force that an object senses in each point in space and time). Given these tight interactions among physical and biochemical processes during morphogenesis, it has become increasingly important to address questions such as: how can mechanical information robustly emerge and contribute to the cellular formation of stereotypical patterns and the regulation of organ shape and growth? how is this information integrated and coordinated along different spatiotemporal scales?

Ingber (2006, 2008) and Mammoto et al. (2013) have suggested that many biological structures can be characterized as a particular type of self-sustained structure that maintains stability by distributing mechanical forces through components that interact via mechanical tension or compression. This energetically efficient architecture appears to permeate structures at the molecular, cellular, tissue, organ and whole-organism levels. The term *tensegrity* was first coined as a contraction of “tensional integrity” and refers to structures that are composed of a network of tensed elements linked to another subset of elements that resist being compressed and, thereby, bring the entire system into a self-sustained state that maintains size and form (Ingber, 2008). A tensegral structure can be visualized as a structure composed of rigid bars and strings; the strings attach to the bars and connect them creating a tensed system that self-stabilizes its shape (Wojtaszek, 2011). In multicellular organisms, the tensional forces applied by cells to the extracellular matrix (ECM) adhesions are balanced by equal and opposite forces such that the shape of tissues is stable (i.e., isometric tension). These forces create a prestressed structural network that can sustain itself and, at the same time, can spontaneously accommodate perturbations (Ingber, 2008). Addition of mechanical energy to this network results in stress channeling through the load-bearing elements and an immediate mechanical responsiveness (Ingber, 2008). In living systems, if stresses are excessive or sustained, the cell, tissue or organ can remodel itself through mechanotransduction (Ingber, 2008; Vermeer et al., 2014). Tensegral systems appear to pervade the organization of living beings. For example, animal cells apply forces to the ECM and tissues reply with equal and opposite forces that stabilize the shape of the tissue (Ingber, 2008). In an analogy with a larger system, Ingber (2008) states that in a human body “the bones that constitute our skeleton are pulled up against the force of gravity and stabilized by the pull of tensed muscles, tendons, ligaments and fascia.”

The tensegral arrangement of organisms and tissues, together with evidence suggesting that some genes and proteins can respond to mechanical stimuli (Mammoto et al., 2012), supports the idea that organismal patterns and shapes partly result from the interplay between internal and external mechanical fields creating a continuum that can communicate cells and organs by long-range information. This type of information can be transmitted along the organism almost instantaneously and without loss of information (actually, the propagation of mechanical signals is faster than the diffusion of a chemical) (see Box 1) (Green, 1996; Ingber, 2008). In the following section we describe how some plant and animal structures can be understood as tensegral systems, and provide examples of developmental patterning processes in which mechanical fields appear to play a central role.

Box 1 **The tensor nature of growth.**

**Tensor fields**

A **tensor** is an algebraic entity that generalizes the concepts of scalar, vector and matrix. Tensors can be considered as *multimatrices*, whose **order** is the number of indices needed to specify its components. For example, a scalar is a tensor of order zero (a single number or quantity specifies a scalar, so no index is needed to define it), a vector is a first order tensor because one needs an index to specify its entries, and tensors of order two can be represented by matrices. Many physical quantities can be expressed as tensors. One example is the body motion under a force. Both the force and the response (acceleration) are vector quantities, so they are related to each other by a tensor (a matrix) that transforms the force vector into the acceleration vector. Plant growth is also an example of a tensor field or simply a tensor, because of its continuous and anisotropic nature. Plant organ and tissue growth can be viewed as the deformation of a continuum, a phenomenon that is studied by elasticity theory (Fung, 1994). Continuum deformation is tensorial and often **anisotropic**, which means that deformations are different in different directions. In general, a material is called anisotropic with respect to a physical property if this property differs in different directions. Otherwise, we say that the material is **isotropic**.

These features, shared by deformation and growth, are both fully described by a tensor (Hejnowicz and Romberger, 1984). Growth can only be described if we know displacement rates in any direction at any given point. It is not possible to provide such a description neither by a scalar nor by a vector. Vectors can specify growth only in the particular direction that they determine, but not in any other. Tensors, on the other hand, can assign a quantity (a growth rate for example) to any given direction at any given point, which is fundamental if one is to describe organ growth. Another important property of a tensor is that it specifies the directions to which maximal and minimal values of this quantity are attained. These are called the **principal directions** of the tensor. A growth tensor describes local changes on an organ during growth and its principal directions are called **principal directions of growth (PDGs)**. (Hejnowicz and Romberger, 1984). Grow rate attains its maximal values on the PDGs of the growth tensor, so it adequately describes anisotropic growth.

Another remarkable property of tensors is that they are independent of the system of coordinates of choice. The root tip growth and the expansion of pollen tubes in plants are examples of morphogenetic phenomena that need a moving coordinate system to be described. Therefore, the use of tensors is essential for the study of symplastic growth.

Two tensor fields are needed to describe growth: **stress** and **strain**. Strain refers to deformation or relative increase in length, and stress is a tensor entity similar to strain, but referred to force (it has units of force/area). Strain and stress are related to one another. According to Green (1996) the macroscopic growth tensor field is the product of three tensors: stress, strain and strength (i.e., resistance to deformation) (Niklas and Spatz, 2012).

**Stress and strain**

The responses of a body to mechanical forces can be described mathematically on the basis of the stress and strain tensors. A body subjected to an external force will undergo deformation or **strain**. The effects of a force applied to a body will of course depend on the dimensions, thickness and geometry of the body. If the force *F* acts on a surface *S* on a body whose area is *A*, then the mechanical stress is defined as

σ=F/A

**Mechanical stress**, or simply **stress**, is thus defined as force per unit area. Stresses are often denoted by σ and a subscript that indicates the specific direction in which the force is acting, so they are adequately described by a tensor. If we consider Cartesian coordinates *(x, y, z)*, and the Cartesian unit vectors *e*_1_ = (1, 0, 0), *e*_2_ = (0, 1, 0), *e*_3_ = (0, 0, 1), then the **stress tensor** is given by the equations

$$\begin{array}{l} \sigma_{\text{x}}=e_{1}\sigma_{\text{xx}}+e_{2}\sigma_{\text{xy}}+e_{3}\sigma_{\text{xz}},\\ \sigma_{\text{y}}=e_{1}\sigma_{\text{yx}}+e_{2}\sigma_{\text{yy}}+e_{3}\sigma_{\text{yz}},\\ \sigma_{\text{z}}=e_{1}\sigma_{\text{zx}}+e_{2}\sigma_{\text{zy}}+e_{3}\sigma_{\text{zz}}, \end{array}$$

**Figure B1.1:**
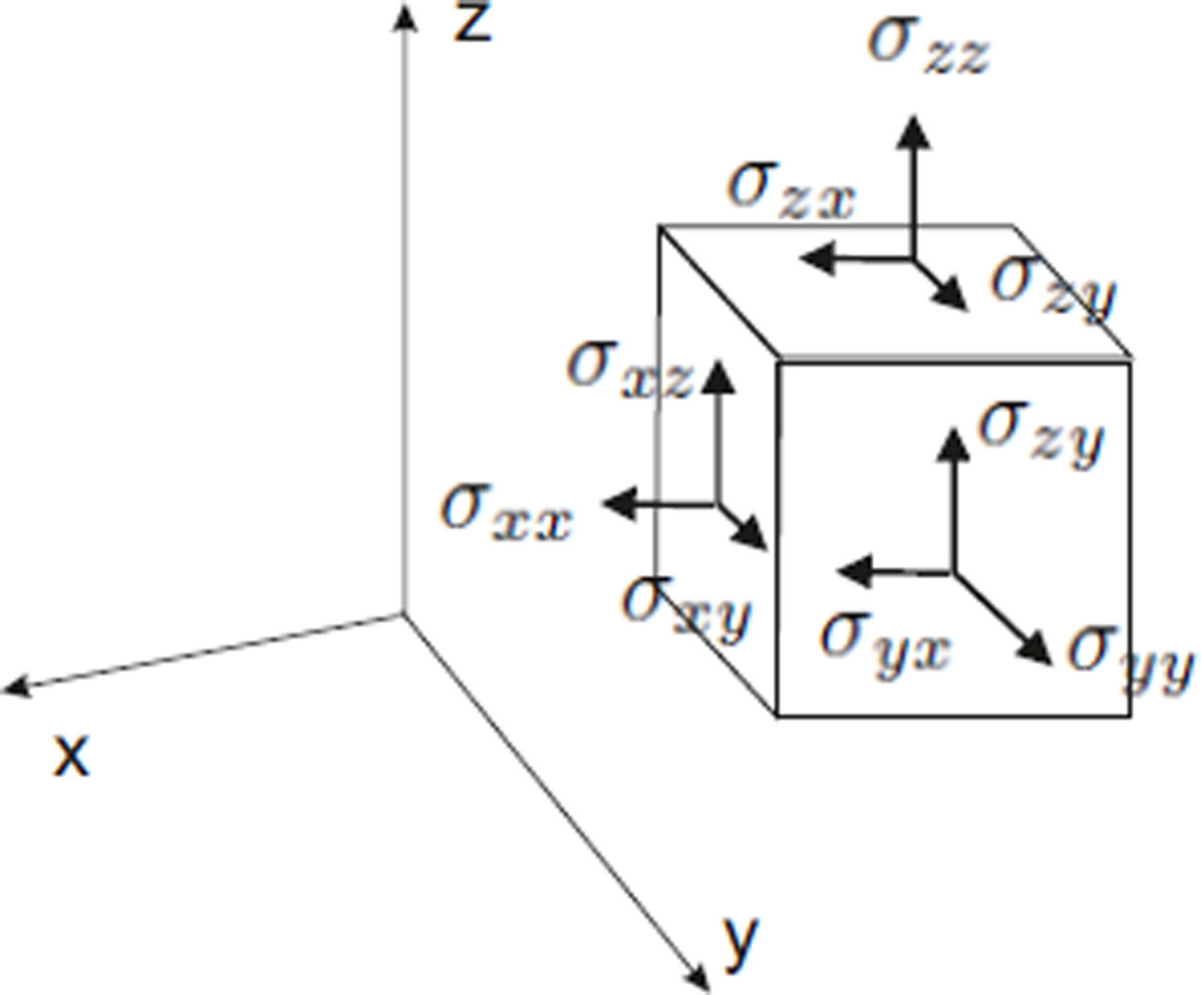
**The stress tensor of a cubic body in Cartesian coordinates**. For each coordinate *x, y*, or *z*, there are three stress components. In this case the normal stresses coincide with the cartesian axes. There always exists a coordinate system in which all tangential stresses are zero, and the nonzero normal stresses are called principal stresses.

The nine components σ_*ij*_ of the stress tensor are shown in Figure B1.1. Note that the components

$$\sigma_{\text{xx}},\sigma_{\text{yy}},\sigma_{\text{zz}},$$

are normal to the surface of the body in the *x, y* and *z* directions, so they are called **normal stresses**. The rest of the components, σ_xy_, σ_xz_, σ_yx_, … are **tangential** or **shear stresses**, as the direction they take is tangential to the body's surfaces. The matrix [σ_*ij*_], *i, j* = *x, y, z*, represents the stress tensor. Thus, by elementary linear algebra (Anton and Rorres, 2004), this matrix can always be brought to a diagonal form, in which all shear stresses are zero. After diagonalization the nonzero elements of the matrix σ_1_, σ_2_, σ_3_, are called **principal stresses** and their corresponding eigenvectors (Anton and Rorres, 2004) are the **principal stress directions**. A positive principal stress is called a **compression**, and a negative one is defined as **tension**. Principal stress directions give the directions in which stress is maximum and minimum. These are of main importance because they allow to fully describe the mechanical state of a body by only three quantities and three directions. The specification of principal directions is the most significant property of a tensor.

**Figure B1.2:**
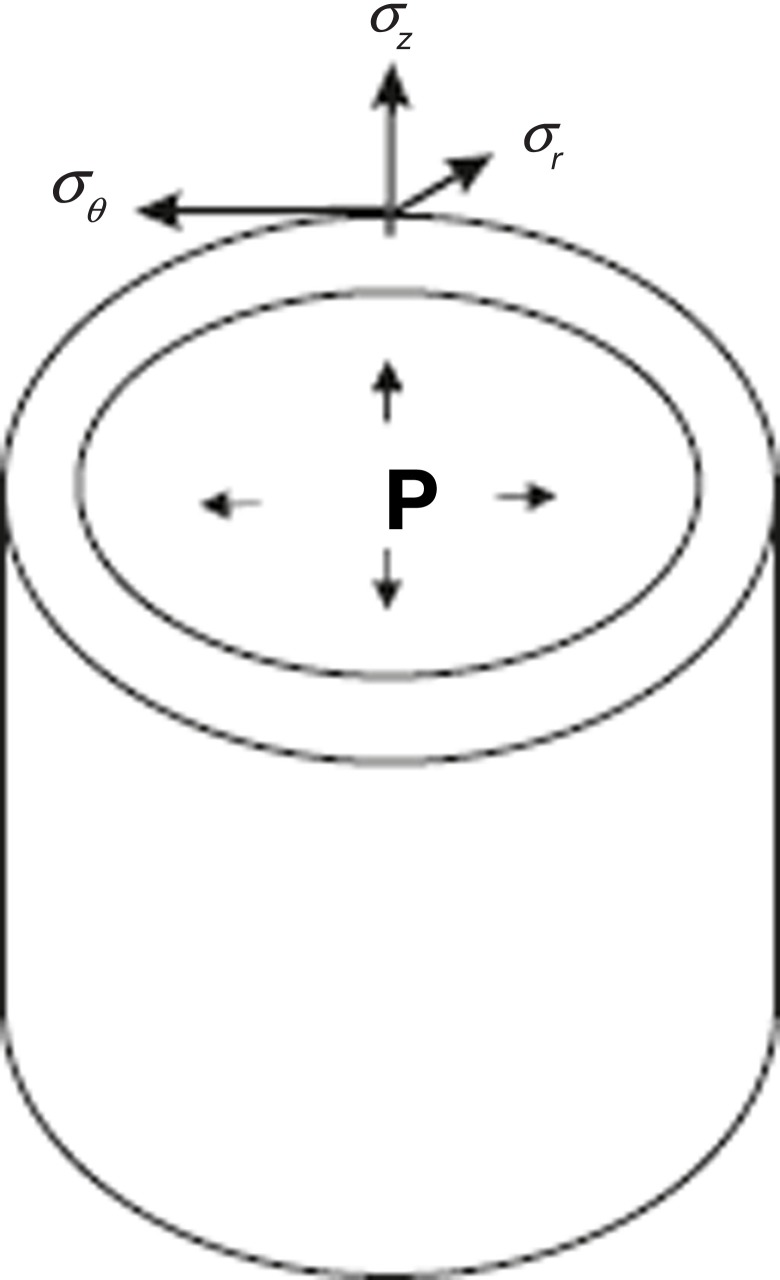
**Principal stresses in a curvilinear coordinate system**. A schematic cylinder subjected to internal pressure *P* is shown. The surface of the cylinder exerts forces due to the pressure *P*, which distributes as stresses in the directions *r, θ, z*. The radial stress, σ_*r*_, is normal to the surface, the stress *σ_θ_* is tangential to the surface and the axial stress σ_*z*_ is in the direction of the *z* axis.

**Principal strains** ε_1_, ε_2_, ε_3_, can be defined in analogous way, in the corresponding principal strain directions. Thus, the tensorial nature of strains makes it possible to describe entire deformation (growth) of a body under mechanical forces as strains along three directions. The shape and geometry of cells, organs and organisms is non-planar, so the study of growth by means of tensors always defines orthogonal curvilinear coordinate systems. Consider, for example, a hollow cylinder under internal pressure P, which can be used to study the expansion/compression of cylindrical structures as stems or vessels. By introducing cylindrical coordinates (*r*, θ, *z*) we can express the principal stresses in the cylinder σ_r_, σ_θ_ σ_z_, due to the pressure *P* as shown in the Figure B1.2. The maximal/minimal stress appears precisely in the radial, tangential and axial directions, so depending on the mechanical properties of the material that constitutes the cylinder, it will deform according to these stresses and directions. In other words, the strain tensor can be defined by means of stress. This is done by formulation of constitutive relations, also called **strain-stress relations**, which describe the response of a material to stress. There exists a wide variety of materials that can be classified into four major types according to their response to forces: elastic, plastic, viscoelastic and fluid.

Elastic materials are the simplest ones in terms of mechanical properties, because the degree of deformation is simply proportional to the applied stress. Elastic materials can store the stress energy and use it to return to its original shape when the force is removed. The stress-strain relation is linear for these materials (they obey the Hooke's law). For an isotropic material this laws can be explicitly given in tensorial form as follows:

$$\begin{array}{l} \varepsilon_{\text{xx}}=1/E\;[\sigma_{\text{xx}}+\nu (\sigma_{\text{yy}}+\sigma_{\text{zz}})],\\ \varepsilon_{\text{yy}}=1/E\;[\sigma_{\text{yy}}+\nu (\sigma_{\text{xx}}+\sigma_{\text{zz}})],\\ \varepsilon_{\text{zz}}=1/E\;[\sigma_{\text{zz}}+\nu (\sigma_{\text{xx}}+\sigma_{\text{yy}})],\\ \varepsilon_{\text{xx}}=(1+\nu )/E\;[\sigma_{\text{xy}}],\\ \varepsilon_{\text{yz}}=(1+\nu )/E\;[\sigma_{\text{yz}}],\\ \varepsilon_{\text{zx}}=(1+\nu )/E\;[\sigma_{\text{zx}}], \end{array}$$

where *E* and ν are important elastic constants known as the **Young's modulus** and the **Poisson's ratio**. The Young's modulus is the slope of the stress-strain curve (in uniaxial tension or compression). It has dimensions of stress (*N/m*^2^) and is a measure of the stiffness of the material: the larger the value of *E*, the stiffer the solid. When a material is subjected to uniaxial compression or tension, it will undergo lateral expansion or contraction. The Poisson's coefficient is the ratio of the magnitude of this two deformations (lateral and axial deformations), and it is a measure of the compressibility of the material. These elastic constitutive relations can be inverted to give stresses in terms of strains.

*Plastic materials* are not so easy to describe, because they often dissipate all the strain energy and cannot recover their initial shape. The constitutive relation is no longer linear and has no standard form. Viscoelastic materials respond to forces by recovering their initial shape only partially when the force is removed. Finally, fluid materials are those which deform continually under stresses. Biological materials show a wide variety of mechanical properties, including those mentioned above and more (Niklas and Spatz, 2012).

## Tensegrity and the generation of mechanical information in animal and plant systems

In eukaryotic cells, the cytoskeleton is a dynamic structure composed of actin filaments, intermediate filaments, and microtubules. It connects the nucleus to the ECM or other fibrous matrices, organizes the cytoplasmic content, guides the transport of molecules from the cytosol to the plasma membrane, and largely determines the form of the cells (Fletcher and Mullins, 2010). The cytoskeleton also senses and rapidly changes in response to contact, pressure or tension, and may transmit this information to the nucleus (Hamant et al., 2008; Ingber, 2008; Mammoto et al., 2013).

**Figure 1:**
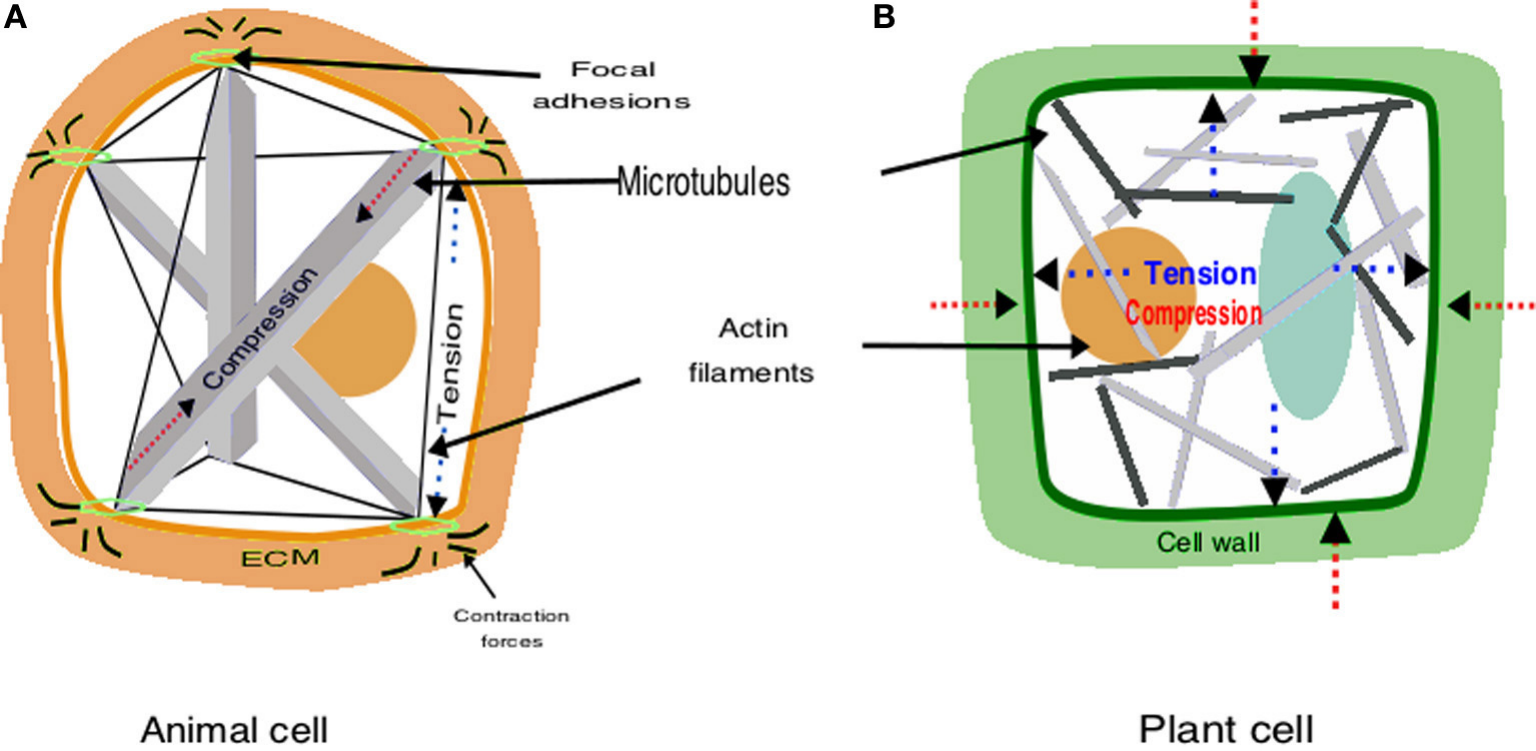
**Schematic representation of tensegrity construction in animals and plants**. **(A)** In animals, the architecture results from the interplay between compressive microtubules and tensile actin filaments; this structure allows to both perceive mechanical signals and to maintain cell shape. **(B)** In plants turgor pressure exerted by the cytoplasma and vacuole (blue) pulls out against cellulose microfibrils, which are tensed; the rigid cell wall gives shape to cells and the cytoskeleton is released from the architectural function.

At the surface of animal cells the cytoskeleton couples to integrins, which are transmembrane proteins that are part of macromolecular complexes called focal adhesions (Ingber, 2008; Wojtaszek, 2011). The intracellular domain of integrins binds to the cytoskeleton via actin-associated proteins such as talin, α-actinin, filamin and vinculin (Ingber, 2008). The extracellular domain of integrins binds to ECM proteins such as fibronectin, laminin, vitronectin and collagen (Baluska et al., 2003). In this manner, the inside of the cells is connected to the outside by a fibrous continuum linking the cytoskeleton, plasma membrane and ECM (Figure 1). However, focal adhesions are not fixed; they are dynamic and respond to mechanical stimuli exerted on the cells. When mechanical stresses are focused on these sites, focal adhesions change their shape and induce the influx of calcium through stress-sensitive ion channels, activate the phosphorylation of proteins and small GTPase pathways, and increase signaling through the cAMP (Mammoto et al., 2004). All these responses can stimulate the transcription of specific genes that in turn may affect the proliferative or differentiation fate of cells. For example, tension application to integrins activates Rho GTPases and its downstream effectors (Mammoto et al., 2004). This signaling cascade results in the regulation of the F-box protein Skp2 that controls the degradation of the critical cyclin-dependent kinase (CDK) inhibitor p27, which regulates the G1/S transition (Mammoto et al., 2004). Then, rather than just anchoring the cell to the ECM, focal adhesions function as mechanosensors that transmit the mechanical state of the ECM to the cell interior (Engler et al., 2006; Wojtaszek, 2011). The dynamics of cell proliferation, in turn, cause changes in the local tension and compression conditions and feedback to the mechanical state of the tissues (Weiss, 1959; Wojtaszek, 2011; Barrio et al., 2013). In this model, contractile actomyosin filaments, and other cytoskeletal components are the major tension elements that winch in the cytoskeleton against tent peg-like adhesions, and microtubules are considered to resist compression and to balance tensile forces (Ingber, 2008; Wojtaszek, 2011) (Figure 1).

Plants also appear to exhibit tensegral structures. Nevertheless, there are two key differences between plants and animals that must be taken into account: instead of the ECM plants have a cell wall that is relatively stiffer, at least when cells are not growing, and have a higher hydrostatic internal force (i.e., turgor pressure). The cell wall is a network of rigid cellulose microfibrils cross-linked by polysacharides and proteins that confer stiffness (Cosgrove, 2005; Wolf et al., 2012). Turgor is a hydrostatic pressure that acts on the cell wall and the plasma membrane. The cellulose microfibrils are the main load-bearing elements of cell walls and are tensed by turgor pressure (Wolf et al., 2012). When turgor pushes outwards cellulose microfibrils respond with an equal and opposite force (Boudaoud, 2010). Plant cell and organ growth are largely based on the balance between these two forces; when the cell wall loosens it yields to turgor, which provides the energy required for cell elongation (Cosgrove, 2005; Boudaoud, 2010). Opposite to the animal model where tensed elements are pulling against compressed ones, in the tensegrity model of plants the compression elements tense the surrounding network (Ingber, 2008). This means that “the tensegrity function fulfilled by the cytoskeleton is replaced by the tensegrity of the cell wall” (Wojtaszek, 2011) (Figure 1).

Several studies suggest that plants also have a cell wall/plasma membrane/cytoskeleton continuum that is functionally comparable to that of animal systems (Wyatt and Carpita, 1993; Reuzeau and Pont-Lezica, 1995; Wojtaszek, 2011). During plasmolysis, for example, cytoplasmic threads and microtubules can be present in Hechtian strands (i.e., stretched plasma membrane extending from the plasmolysed protoplast to the cell wall) (Lang-Pauluzzi and Gunning, 2000). In agreement with this idea, there is a tight coupling between the mechanical stress of the cell wall and the spatial orientation of microtubules (Hamant et al., 2008; Uyttewaal et al., 2012). Indeed, in both plants and animals Rho GTPases and Rho of plants (ROP)-GTPases, respectively, control spatial cellular processes by signaling to the cytoskeleton and vesicle trafficking (Szymanski, 2009; Nagawa et al., 2010; Wojnacki et al., 2014).

Some evidence supports the involvement of integrin-like proteins in plants (Swatzell et al., 1999). However, no true integrin homologs and actin-associated proteins that link integrin to actin cytoskeleton have been found (Baluska et al., 2003; Monshausen and Gilroy, 2009). Several molecules have been proposed for connecting the plasma membrane to the cell wall: formins, wall-associated kinases (WAK), cellulose synthase (CESA) complexes, receptor-like kinases (RLKs), and arabinogalactan proteins (AGPs) (Reuzeau and Pont-Lezica, 1995; Baluska et al., 2003; Monshausen and Gilroy, 2009; Wojtaszek, 2011). Nevertheless, none of these molecules have been directly implicated in mechanical responses.

Since plant cells do not migrate, morphogenesis in plants is largely determined by the regulation of the local rate and direction of cell growth and proliferation. The mechanical state of the extracellular medium is thus central in the generation of such patterns and the coupling between CESA complexes and the cytoskeleton appear as key in this process; they couple the cell's interior and the cell wall, and it has been shown that the anisotropic growth rate is larger in the direction perpendicular to the orientation of cellulose microfibrils in the wall (Hamant et al., 2008; Uyttewaal et al., 2012). Microtubules orient parallel to the maximal tension axis and guide the deposition of CESA complexes that, in turn, locally reinforce the cell wall (Wymer et al., 1996; Paredez et al., 2006; Hamant et al., 2008). Actually, when the competence of cells to respond to tension is lowered or the interaction between CESA complexes and microtubules is impaired, normal growth is affected (Uyttewaal et al., 2012; Landrein et al., 2013). For example, the disruption of microtubule-guided cellulose deposition leads to torsion of several plant organs and new phyllotactic patterns (Ishida et al., 2007; Landrein et al., 2013). According to this, the mechanical information is a source of variability with important implications for the creation of diverse living forms during development and the subsequent processes of evolution (Niklas and Kutschera, 2012).

## Examples of the role of mechanical information in the formation of periodic structures in plant and animal development

### Mechanical forces as position-dependent information in the periodic formation of organs in plants

Auxin is a plant hormone that is central for plant development. Among the various processes in which auxin participates are the periodic formation of plant organ primordia, cell elongation, and cell proliferation. The patterns of auxin concentration are associated to the cellular organization along the root meristem, the periodic formation of shoot buds, or the formation of lateral roots (Zažímalová et al., 2014). Auxin is moved throughout the plant by means of a particular system of polar transport. The auxin efflux carriers PIN-FORMED (PIN) are preferentially localized in regions of the cell plasma membrane, thus polarizing auxin fluxes. In turn, the position of PINs in the membrane correlates with auxin fluxes (Wiśniewska et al., 2006). In the shoot apical meristem (SAM) of *Arabidopsis thaliana*, PIN1 is directed toward the neighboring cells with auxin maxima (Reinhardt et al., 2003). This positive feedback depletes auxin in the cells that are close to auxin maxima and inhibits the formation of new organs around the emerging primordia, yielding the observed phyllotactic and rizhotactic patterns. The cellular polarization of PIN proteins is dynamic in response to environmental signals (e.g., gravitropism) (Rakusová et al., 2011) and involves the regulation of trafficking intracellular vesicles that modulates the rates of auxin efflux (Dhonukshe et al., 2008). Moreover, auxin inhibits the internalization of PIN proteins (Paciorek et al., 2005; Nagawa et al., 2012) and regulates its expression (Vieten et al., 2005).

Several molecular elements regulate the endo- and exocytosis of these proteins (Dhonukshe, 2012). There has been a debate about the mechanisms underlying PIN polarization. Some authors hypothesize about a flux sensor component (Mitchison, 1980), while others argue that cells perceive the concentration of auxin in neighboring cells (Smith et al., 2006). Interestingly, in postembrionic development, auxin spatiotemporal distribution has been shown to affect and respond to physical forces such as the mechanical tension of the plasma membrane (Heisler et al., 2010; Nakayama et al., 2012). When the cells shrink or swell there is a change in the surface area of the plasma membrane given by the retrieval and delivery of membrane materials (Homann, 1998). It then seems that PIN proteins are directed to the areas of maximum tension (Heisler et al., 2010), suggesting that the rate of endo- and exocytosis, and thus the deposition of molecules such as PINs, depends on the mechanical tension of the membrane. In agreement with this idea, PIN1 density at the plasma membrane, and the concomitant auxin concentrations, respond to induced swelling or shrinking in tomato cells (Nakayama et al., 2012).

It has also been suggested that the mechanical state of the cell wall affects the polarization of the PIN proteins (Fleming et al., 1997; Feraru et al., 2011; Braybrook and Peaucelle, 2013). The acidification of the cell wall enhances the activity of several enzymes, such as expansins and pectin methyl esterases that, in turn, enhance the elastic properties of the cell wall (Fleming et al., 1997; Sánchez-Rodríguez et al., 2010; Peaucelle et al., 2012). Several studies indicate that auxin, which is an acid, changes cell wall pH and cell wall rigidity (Cleland, 1971; see a review in Hager, 2003). Additionally, it has been shown that changes in the position of PINs within the cell, and thus auxin fluxes, are associated to rapid changes in the orientation of the microtubule cytoeskeleton (Heisler et al., 2010). Both changes in microtubule orientation and PIN localization can be induced by local perturbations, such as the ablation of neighboring cells, or local changes in the cell wall mechanical properties (Paredez et al., 2008; Heisler et al., 2010; Braybrook and Peaucelle, 2013).

**Figure 2:**
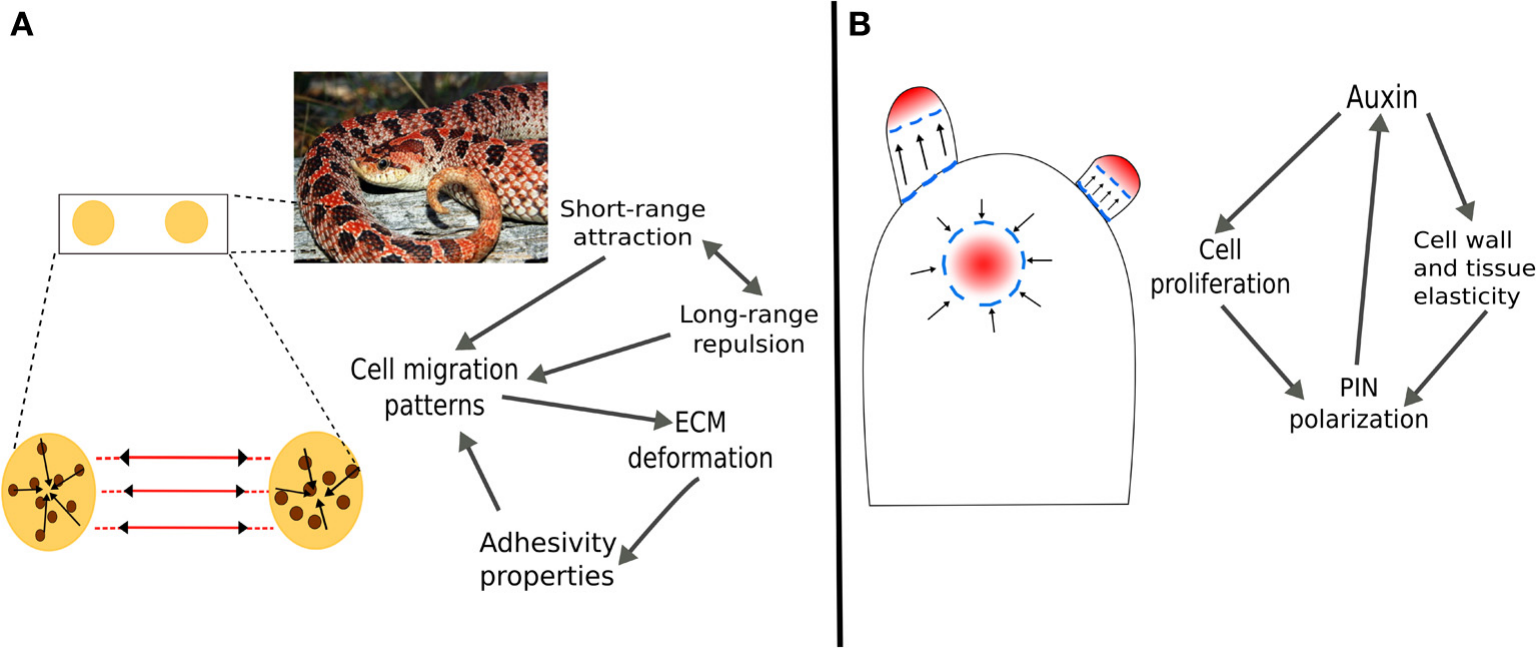
**Mechanical forces as positional dependent information in the formation of periodic structures in plants and animals**. **(A)** In vertebrates, the formation of pigment patterns is determined by attraction/repulsion of chromatocytes and the deformation of the mesenchyme that generate tension tracks through which cells migrate. **(B)** In plants, the enhancement of cell wall and tissue elasticity by auxin creates undulations at the SAM surface. Furthermore, auxin regulates genetic programs that promote cell proliferation and differentiation into the different organ primordia. The modification of the mechanical field serves as positional information for the polarization of the PIN auxin efflux transporters. In both examples, long-range forces caused by changes in the mechanical field have a delimited range of action which is indicated by the periodicity of patterns.

The phenomenology described above could be integrated under the umbrella of the tensegrity concept. Cell wall fibers and microtubules may be considered as part of a tensegral system (Figure 2) in which microtubules would correspond to relatively more flexible elements that can spontaneously react to and accommodate changes in the mesoscopic mechanical field. With reorientation and rearrangement of microtubules, changes in the mechanical fields can be transmitted almost instantly to changes in the distribution of forces within the cell and its membrane. The newly generated points of maximum tension in the membrane can cause the differential distribution of vesicle cargo, such as auxin transporters PINs and the concomitant modification of auxin fluxes. As detailed above, in the longer term, auxin fluxes and the presence of some enzymes can affect the local mechanical properties of cell walls, which would feedback mechanical fields and also generate microtubule arrangements that reinforce or stabilize local anisotropies and spatial patterns in cell shape and growth.

Parallel to the tensegrity structure of cells, plant tissues and organs are mechanically integrated. At the organ level, tissue stresses that result from turgor, proliferation dynamics, structural variation of tissues, etc., create a tensional integrity. Outer tissues impose a mechanical constraint to the expansion of internal tissues (Kutschera and Niklas, 2007). Therefore, outer tissues are tensed and internal tissues are compressed, meaning that the organ is also a tensegral structure. The prestress created is a necessary condition for several morphogenetic processes, for example, the buckling of the SAM surface during phyllotaxis (Wojtaszek, 2011). It has been postulated that, when the elasticity of the cell walls at the SAM surface is enhaced, inner tissues provide the driving force to create discrete undulations without any prepatterning (Wojtaszek, 2011). The long-range forces that result from these undulations could serve as positional information for the creation of auxin maxima and, hence, the spacing of organs during phyllotaxis. These mechanical processes are coupled with biochemical and genetic dynamics to yield the emergent patterns of organ primordia (Newell et al., 2008).

### Mechanical forces as position-dependent information in the formation of pigment patterns in vertebrates

The emergence of pigment patterns in fishes, reptiles, mammals and other vertebrates has fascinated researchers and has been the subject of embryological, genetic, mathematical and other types of studies. For instance, Turing-like systems assume the existence of morphogens that, by simultaneously diffusing and reacting, can generate heterogeneous concentration patterns resembling those of animal skins. It has also been postulated that patterning mechanisms equivalent to these reaction-diffusion systems can emerge also from interactions with gene regulation and cellular communication (Kondo and Miura, 2010).

Pigment pattern formation involves the arrangement of epithelial sheets and cells during early stages of embryogenesis (Schock and Perrimon, 2002). During this process, pigment cells migrate on the mesenchyme, a fibrous matrix with biphasic (consisting of both solid and liquid fractions) and viscoelastic (exhibiting viscous and elastic properties when deformed) properties that can show both tension and compression forces (Grinnell and Petroll, 2010). It has been shown that cells embedded in a fibrous matrix can deform it in a way such that the matrix fibers are reoriented into tension lines. Grinnell and Petroll (2010) review the mechanisms involved in the adhesion and migration of cells embedded in a viscoelastic matrix, and mention that cell traction can deform viscoelastic tissues by establishing adhesive interactions and locally contracting the underlying matrix. These interactions between epithelial cells and the mesenchyme matrix modify the mesoscopic mechanical field. In turn, the long-range forces that result regulate cell migration and establishment; the tension lines serve as tracks for cell migration and accumulation (Weiss, 1959; Caballero et al., 2012). Together, the long-range forces that result from mesenchyme deformation and the reported attraction/repulsion between different and similar types of chromatocytes, have been proved sufficient to generate distinct color patterns in vertebrates (Caballero et al., 2012). This mechanism is consistent with experimental evidence and couples molecular and physical processes and provides a conceptual framework to study morphogenesis from a tensegrity-based perspective.

Moreover, this mechanism may help address another fundamental problem in developmental biology, namely, how the size and spacing of organs and anatomical structures is controlled during development. While local cell–cell interactions and unbounded morphogen diffusion are not sufficient to explain this type of controls, mechanical forces are bounded or have a delimited range of action, as evidenced by the periodicity of patterns in animal and plant bodies; the end of one pattern period and the beginning of another indicates the characteristic length of the long-range forces.

As for the plant case, the notion of tensegrity helps clarify and integrate the phenomena described above. While the animal cell itself appears to follow tensegral principles (Ingber, 2008; Figure 1), the mesenchymal-chromatocytes pattering system can be understood as a tensegral system that goes beyond the cellular scale. It is conformed of fibrous elements of the mesenchyme and cytoskeletal fibers located inside the cell. Both types of fibers are connected via focal adhesions and transmembrane proteins that respond to force changes on both sides of the membrane (Schock and Perrimon, 2002), thus creating a mechanical coupling that transfers the tension generated within the cytoskeleton to the mesenchymal matrix and neighboring cells. Because the suggested tensegral system is in a prestressed state of tension, a change in the matrix force fields also causes a realignment of structures within the cytoplasm and the corresponding change in cytoeskeletal arrangement, cell function, and the velocity of cell migration (Weiss, 1959; Ingber, 2008). Similarly, changes in cytoskeletal tension generated by the action of actomyosin motors and polymerization of microtubules is transferred to the matrix fibers and distributed in the whole tissular scale (Ingber, 2008). Then, as chromatocytes migrate and adhere to the surrounding matrix, they remodel the fibers and tension fields in the mesechyme, which then promote the movement and adhesion of further migrating cells on the regions of highest fiber density. Due to the nested tensegral systems ranging from the cellular to the organismal scale (Lakes, 1993), all these changes can occur spontaneously and rapidly, and result in stereotypical patterns constituting positional information.

We have focused on the similarities between plant and animal tensegral structures, yet it is worth mentioning that plant and animal cells differ in important features. Since plant cells have a cell wall, these are often more rigid than animal cells during embryogenesis. However, animal cells in adult tissues are surrounded by a rather rigid matrix, while the cell walls of proliferating and growing cells loosens and are relatively flexible during postembryonic development. Then, the elasticity of the fiber arrangements that conform both plant and animal tissues changes considerably during development, and might even have similar characteristics in animal embryos and developing regions of a plant. Indeed, the capacity of cells and organisms to change their material properties through growth and development confers spatial and temporal heterogeneity on the mechanical behavior of the organisms' body and constituent parts (Niklas and Spatz, 2012).

## The mechanical isotropy hypothesis for the generation and maintenance of stem-cell niches in multicellular organisms

Along this text, we have argued that mechanical forces acting on tensegral structures formed by cells and tissues, coupled with molecular mechanisms that regulate, feedback or respond to these forces, may generate spatially dependent information relevant for development. We then provided some examples briefly illustrating how these forces may lead to the formation of periodic patterns arising in tensegral structures. From our current understanding of these and other model systems, one might suggest some mechanical principles shared by developing organisms from diverse lineages. Here we revisit previous ideas in this direction and hypothesize that there are important structural similarities in the organization of the pools of undifferentiated cells (stem cells) that give rise to all the differentiated cells and tissues in plants and animals, and that the specification of such cells emerges, at least in part, from the interaction between cellular dynamics and generic mechanical forces.

**Figure 3:**
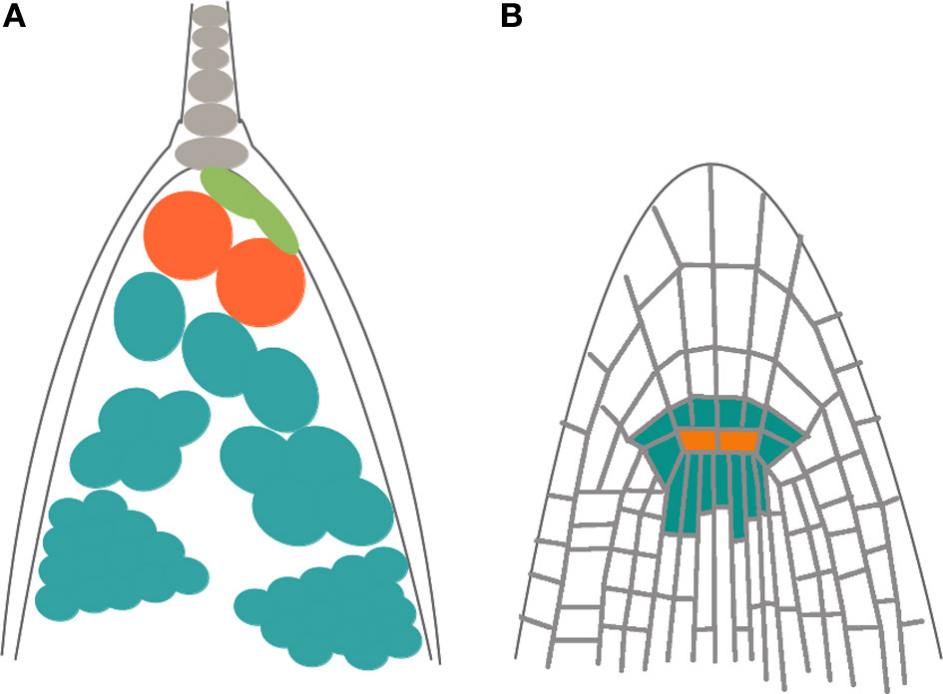
**Schematic representation of the structural similarities in the organization of stem cell niches (SCMs) in plants and animals**. **(A)** *Drosophila melanogaster* ovary, and **(B)** root apical meristem (RAM) of *Arabidopsis thaliana*. In both cases, organizer cells (orange) are surrounded by pluripotent stem cells (blue) that divide rapidly and that, after a determined number of divisions, elongate, and acquire a particular cell fate.

In both plants and animals, stem cells are maintained in a particular environment known as stem-cell niche (SCN), which is conformed by the so-called organizer cells surrounded by multipotent stem cells (Scheres, 2007). As other authors have noted (Sablowski, 2004; Scheres, 2007), animal and plant stem cell niches are structurally similar; in both cases pluripotent stem cells are located around or next to a few organizing quiescent cells. Also, in both systems stem cells give rise to rapidly dividing cells that after a determined number of divisions begin to acquire a particular cell fate. Additionally, the plant and animal SCNs that have been thoroughly studied and now constitute classic models (e.g., the *Drosophila melanogaster* ovary, and the mammalian gut and hair SCNs in animals; the shoot and root apical meristems in *A. thaliana*) are located in tubular structures close to concave surfaces (Figure 3).

While the genetic and biochemical elements associated to SCN organization do not seem to be overall conserved in plants and animals (Sablowski, 2004; Scheres, 2007), we speculate that some of the structural aspects shared by these systems arise from common mechanical principles and the interactions among physico-chemical fields and regulatory networks. Indeed, some general features of SCNs may also be attributed to similarities in the structure and dynamics of the biochemical networks or regulatory motifs associated to SCN maintenance, even if the elements of these networks are not the same (Sablowski, 2004; Azpeitia et al., 2010; Azpeitia and Alvarez-Buylla, 2012), except for some that are also conserved such as the *RETINOBLASTOMA* gene (Sablowski, 2004). Specifically, we revisit the idea that the position and *stemness* of cells within plant and animal SCNs is partly determined by mechanical properties associated to the geometry of the organ containing them and the relationship between compression and tension forces acting on the cells. Indeed, as we detail and illustrate below, animal and plant SCNs appear to be located in critical stress points in which tension and compression forces, to which cells are subjected, converge (Wojtaszek, 2011). This hypothesis has been put forward for animal stem cells along with the notion of *force isotropy* (i.e., when forces exerted by the cell or the adhesion substrate have the same magnitude in different spatial orientations) (Nava et al., 2012), and has also been postulated for the plant case (Lintilhac, 1974a,b; Wojtaszek, 2011).

**Figure 4:**
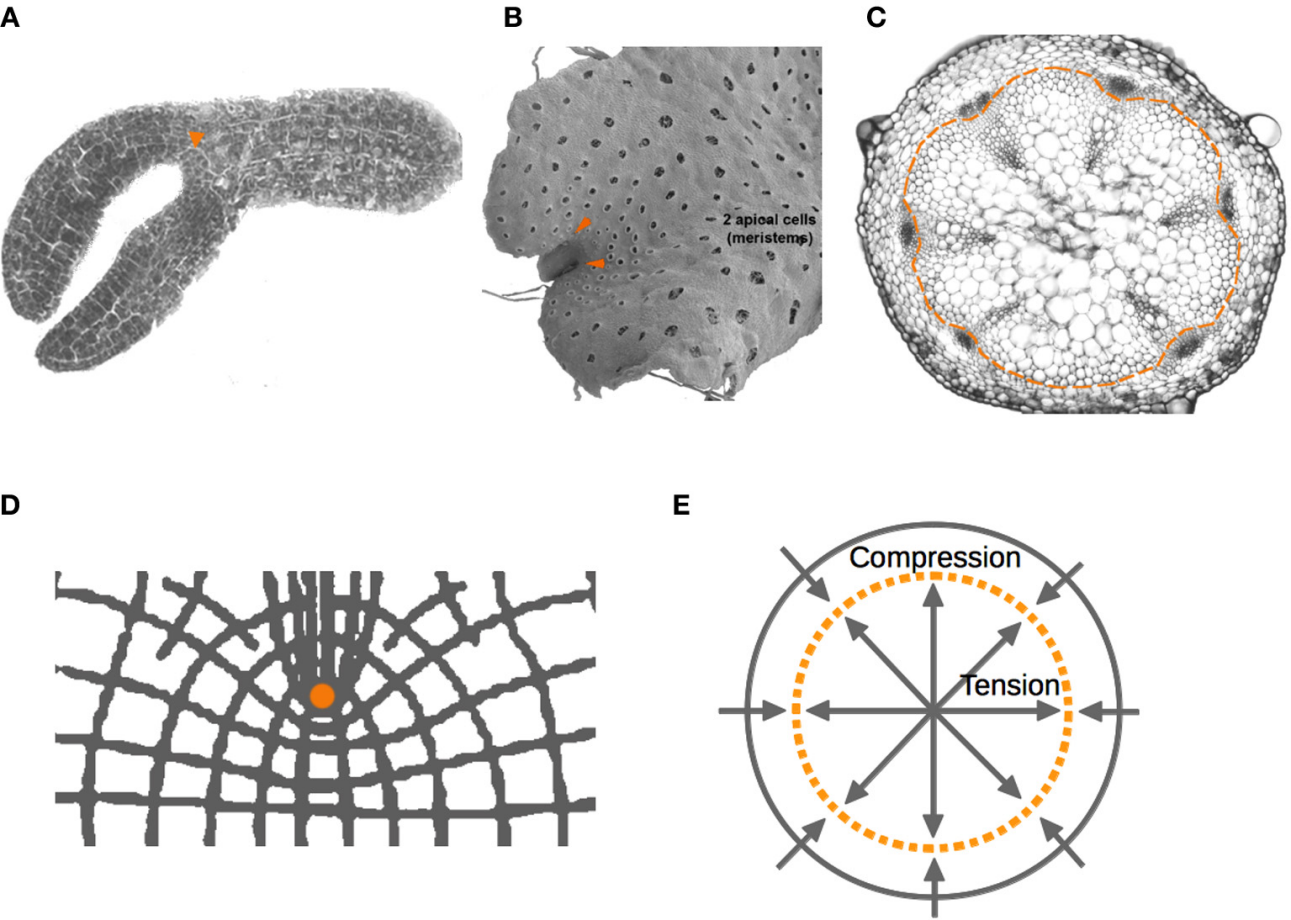
**Localization of plant meristems**. Localization of plant meristems. Apical and axillary meristems (arrowheads) of **(A)** *Arabidopsis* embryo, and **(B)** *Marchantia* gametophyte, respectively. **(C)** Vascular meristems that generate xylem and phloem tissues during radial growth are located within a narrow ring (dashed line). **(D)** Lintilhac (1974a,b) showed the points of stress concentration generated by notches. He also predicted that ring-like geometries (dashed line) would be zones where forces of tension and compression nullify **(E)**. We hypothesize that the mechanical properties of these regions are part of a potentially generic mechanism for the localization and maintenance of SCNs in multicellular organisms.

As postulated by Lintilhac (1974a,b), the principle of shear-free partitioning states that, in a plant cell under tension and compression, new cell plates will form in the plane that is free of shear stresses, perpendicular to the axis of applied stress. Plant cells often grow anisotropically in the direction perpendicular to the cell plate (Green, 1962), then, in actively dividing plant tissues the stress will be reinforced by enlargement of the cell, thus inducing the same orientations of new walls in daughter cells and perpetuating the cell division pattern (Lintilhac, 1974a,b) (Box 2). This mechanism constitutes a generic and relatively simple way of initiating and propagating an apex and can explain the maintenance of an apical tip itself. Key to this proposal is that, depending on force relations on the tip, the growing apex may adopt either of two characteristic forms: a concave apex (e.g., apical meristems in plants) or a convex apex (cardioid-like meristems) (Figure 4). Lintilhac (1974a) used the Lamé-Maxwell equations of equilibrium within a two-dimensional elastic body to locate the point of mechanical isometry in a concave or a convex domain (Box 2). He suggested that these concave or convex plane domains could represent a two-dimensional section of an apical or some axilar plant meristem, respectively, and found the mechanically isometric points. Cells located on or near these isometric regions must then have particular properties in terms of division rate (Lintilhac, 1974a,b). Other authors have also noted that this isometric condition entails particular modes of cell-to-cell communication (Oparka and Prior, 1992), as well as specific gene expression patterns (Chen et al., 1997).

Box 2 **Free planes and mechanically isometric points.**

**Shear-free plane**

A plane in three-dimensional space is completely determined by its normal vector, that is the vector which is perpendicular to the plane (Anton and Rorres, 2004). The three principal directions of stress (see Box 1) in a loaded body define three distinct planes, each of one is determined by the direction to which it is normal. As the principal stress directions are mutually orthogonal, a plane that is normal to one of them must contain the two orthogonal vectors that define the other two directions. Such a plane is called **principal plane**. In a real three-dimensional body under tension or compression, principal stresses and principal planes can be determined experimentally (Heywood, 1969). Thus, it is possible to define principal planes in real three-dimensional structures that do not exert shear stress. They may also be called **shear-free planes**. One of these planes often coincide with cell division planes, according to the observations of (Lintilhac, 1974a; Lynch and Lintilhac, 1997). This is clearly seen in anisotropic growth: for isotropic growth planes of cell division are not related to strains or stresses because they are the same in all directions, so these planes appear to be randomly oriented. However, when growth is anisotropic the directions of maximum and minimum stress differ, and reinforcement of the cell walls in the direction of maximal tension is present (Green, 1996), leaving the remaining principal directions available for growth.

**Figure B2.1:**
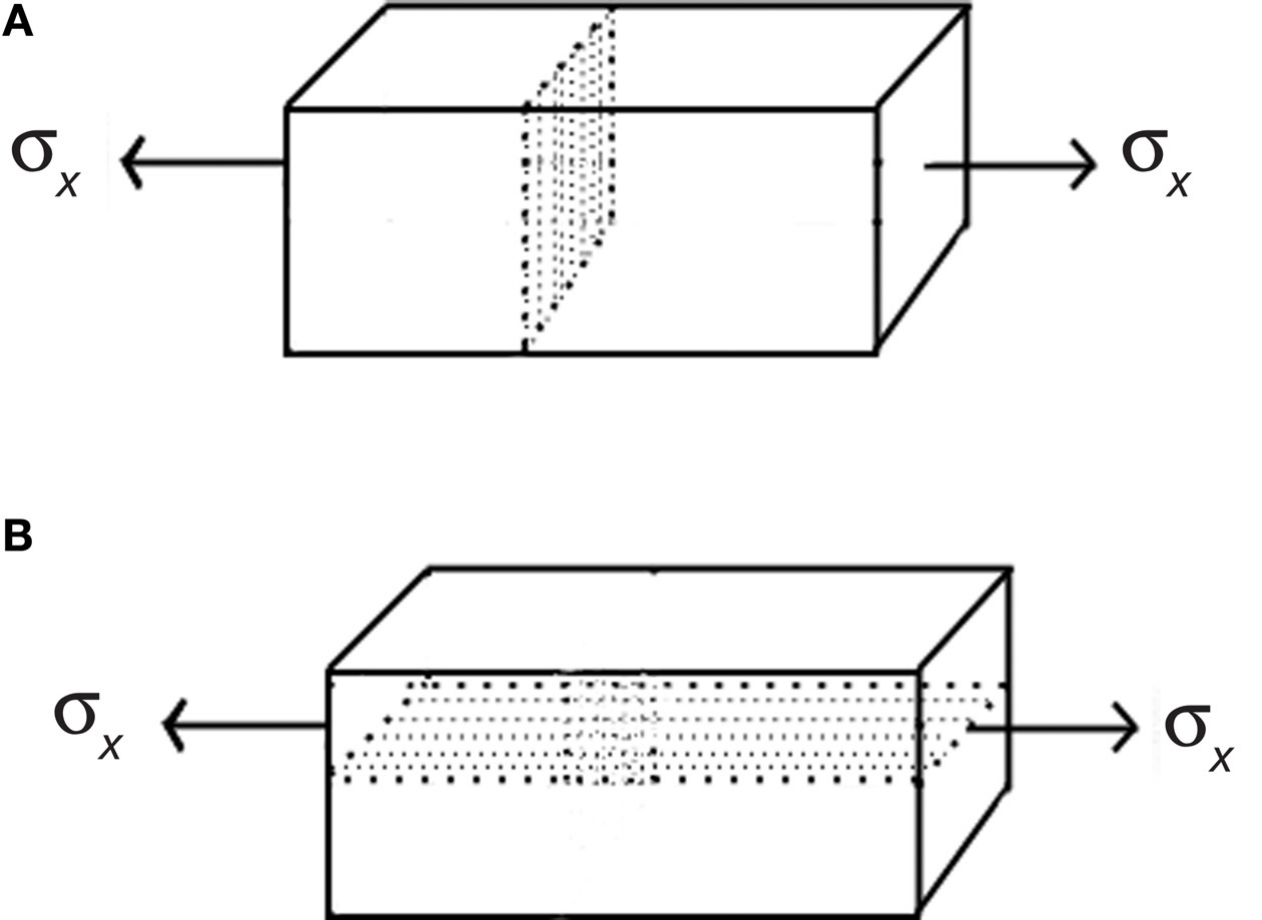
**Principal planes. (A)** A solid body subjected to uniaxial tension and the principal plane, which is parallel to the direction of applied force. All tangential or shear stresses are zero along this plane. **(B)** The plane that is perpendicular to the principal plane undergoes the maximal shear stresses generated by uniaxial stress σ_*x*_.

The shear-free plane is easy to determine in the case of uniaxial stress, because it is the plane perpendicular to the applied force. Because the stress tensor is diagonal, the shear stress is zero in the planes of the stress tensor and principal stresses (see Box 1). On the other hand, the plane that is perpendicular to the shear-free plane exerts the maximum shear stress. Figure B2.1 shows the shear-free plane in A, perpendicular to the applied stress σ_x_ in the x direction, and the maximum shear stress plane in B. If the cell divides in the shear-free plane, the state of stress of the daughter cells will be the same as the original one, so they will be subjected to uniaxial tension (or compression) and their shear-free planes will locate in the same direction as their mother's free plane. The inheritance of the shear-free plane will thus perpetuate the cell division pattern.

**Lamé-maxwell equations**

The stress state at a point in a two dimensional case is completely determined if the stress components on any two perpendicular planes passing through the point are known. Principal-stress trajectories are lines that are tangent to the two principal stresses at any point. Since the principal stresses are mutually orthogonal, these stress trajectories form orthogonal families of curves. Lamé-Maxwell equations express the stress equilibrium state of a body by using the principal-stress trajectories. In a Cartesian coordinate system (x,y) one can obtain the equations of stresses in equilibrium by performing the summation of all moments of forces acting on the body in the *x* and *y* directions and setting them to zero. These are the well-known stress equilibrium equations:

$$\begin{array}{l} \partial \sigma_{\text{x}}/\partial \text{x}+\partial \tau_{\text{xy}}/\partial \text{y}=0,\\ \partial \tau_{\text{xy}}/\partial \text{x}+\partial \sigma_{\text{y}}/\partial \text{y}=0, \end{array}$$

where σ_x_ and σ_y_ are normal stresses and τ_xy_ represents shear stress.

However, in some cases it is necessary to use a curvilinear coordinate system. The Lamé-Maxwell equations are useful to express equilibrium conditions in two-dimensional curvilinear coordinates. They are defined in terms of principal stresses and principal stress trajectories S_1_ and S_2_. Let σ_1_ and σ_2_ be the principal stresses in a 2-dimensional curvilinear coordinate system, and let ρ_1_ and ρ_2_ be the radii of curvature of a curvilinear surface element. By equating to zero the sum of all forces parallel to the corresponding principal directions one obtains the Lamé-Maxwell equilibrium equations:

$$\begin{array}{l} \partial \sigma_{1}/\partial S_{1}+(\sigma_{1}-\sigma_{2})/\rho_{2}=0,\\ \partial \sigma_{2}/\partial S_{2}+(\sigma_{1}-\sigma_{2})/\rho_{1}=0. \end{array}$$

These equations illustrate that principal stress magnitudes are intimately related to the shape of stress trajectories in a loaded body. Any discontinuity of such trajectories must be associated with rapid changes in stress magnitude, because the discontinuity distorts the stress trajectory.

**Figure B2.2:**
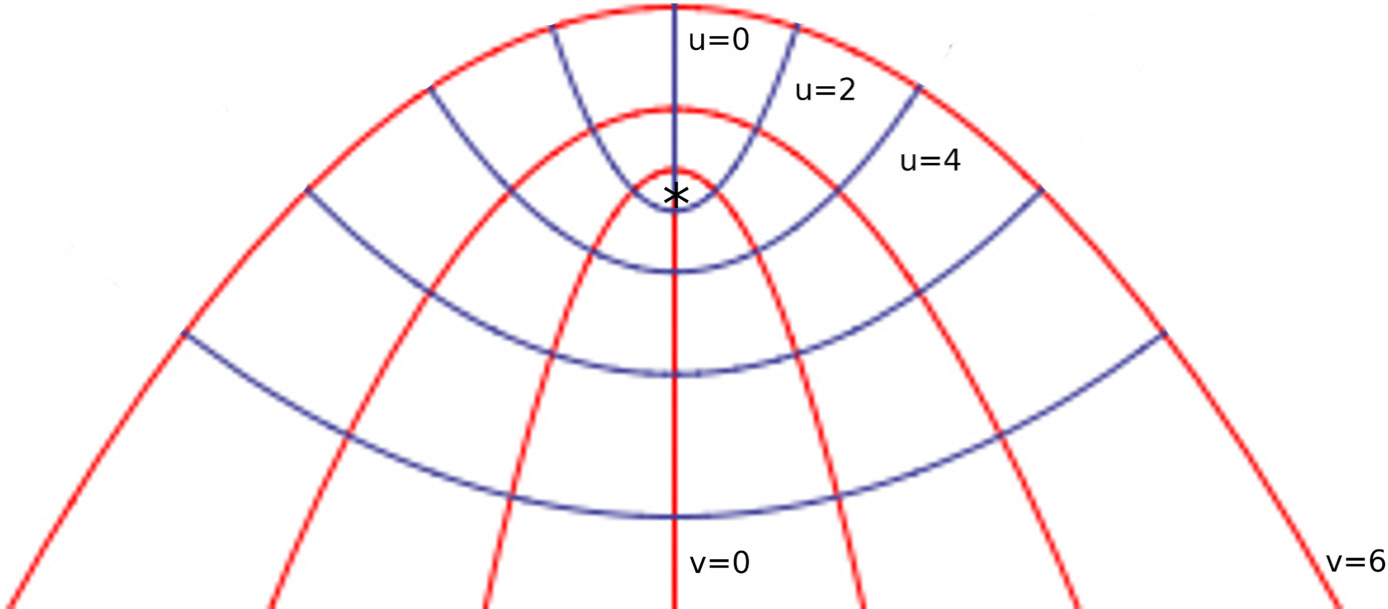
**Confocal coordinate system modeling an apical dome**. Periclines are the red curves corresponding to the *v* coordinate and anticlines correspond to the *u* coordinate. The curved boundary of the surface is represented by the curve *v* = 6, which is under tension produced by internal pressure. The singularity of the system is marked by an asterisk. The curves *(u, v)* coincide with stress trajectories dictated by the Lamé-Maxwell equations on a surface subjected to compression. In this case, the *v* curves are the compression trajectories. The singularity corresponds to the unique region on the surface at which growth rates are nearly zero, meaning that stresses are also almost null at this point.

According to Lintilhac (1974a; Lynch and Lintilhac, 1997), if division planes coincide with shear-free planes, then the principal stress directions could be determined in a growing organ by continuously changing the direction of the normal vectors at division planes. Following the trajectories described by principal directions one gets the natural coordinate system used to study growth patterns of plant organs under stress produced by turgor pressure (Hejnowicz and Romberger, 1984). Hejnowicz and Romberger developed the concept of a growth tensor (strain tensor) by studying the growth rate patterns on different plant structures and defined the principal directions of growth as the principal directions of this tensor (see Box 1). They applied these concepts specially in root and shoot apices, which can be viewed as axisymmetric dome-like structures. Two kinds of trajectories can be distinguished: meridional (periclines) and latitudinal (anticlines) (Figure B2.2). As these trajectories come from the principal directions of a tensor they intersect at right angles and form a curvilinear orthogonal system of coordinates (*u, v*) named confocal coordinates. The third dimension is obtained by rotation about the axis *u* = 0 and *v* = 0 (Figure B2.2), with all periclines and anticlines surrounding the focal point (marked by an asterisk), which is the unique critical point of the system (Hejnowicz, 1984): no addition of more trajectories will produce any other point with the properties of the focus (Hejnowicz, 1984; Hejnowicz and Romberger, 1984; Kwiatkowska, 2004). This focus represents a point of mechanical isometry, where stresses are the same in all directions and growth rates are nearly zero. A clear example of an apical meristem with confocal coordinates is the root apical meristem in which the quiescent center (Clowes, 1961; Kwiatkowska, 2004) coincides with the focal point. Indeed, quiescent center is the region of the root meristem where growth rates are nearly zero in all directions (Nakielski and Barlow, 1995). These trajectories are defined when the growth is anisotropic; for isotropic tissues all directions can be considered as principal (Kwiatkowska, 2004). According to the Lamé-Maxwell equations, in a convex surface subjected to compression, compression stress trajectories follow the profile of the surface boundary, whereas tension trajectories must be perpendicular, as it happens in the confocal coordinates; the mechanically isotropic point locates in the focus of all the paraboloidal trajectories (Lintilhac, 1974a). Thus, the stress trajectories on a curved surface under compression that can be described by confocal coordinates coincide with the strain trajectories on the same surface subjected to tension that results from turgor pressure. These equations and isometric points can be obtained in other curved surfaces under tension or compression.

Symplastic growth is an exceptional feature of plant organs that is crucial when quantifying growth by applying the continuum theory of deformation. This approach seems to be adequate to study mechanical influences on plant morphogenesis. It is unlikely that intracellular detailed mechanisms are necessary to address global processes as the emergence of shape and size and its dynamics, which are mesoscopic phenomena controlled by global constraints (Harold, 2005).

Interestingly, under a growing concave edge, this isometric region is predicted to be around a point where stem-cell niches appear to be stereotypically located, often fixed there by cap cells (Figure 3). Indeed, organizing cells in SCNs are highly symmetrical (more similar to spheres or cubes) in comparison to other cells around the niche, revealing the nearly neutral effect of mechanical forces acting on them. Also, in agreement with this hypothesis, it has been shown that microtubules, whose orientation correlates with the direction of the principal stress, are oriented on average in all directions—isotropically—in the region corresponding to the shoot apical stem-cell niche of *Arabidopsis*, whereas microtubules of cells outside this region are aligned anisotropically on the principal stress directions (Hamant et al., 2008; Heisler et al., 2010; Nava et al., 2012). In the case of a convex curve forming a notch, the same mechanism would generate points of mechanical isometry that appear to correspond to the position of SCNs in cardioid geometries, such as the *Arabidopsis* embryo in the heart stage, in which the shoot SCN is initially established, as well as the local geometry of some axillary meristems. Examples outside vascular plants lead us to speculate that this might be a generic feature of plant meristems, which are located next to notches in plants like ferns and hepatophytes (Figure 4).

It is interesting to notice that besides the apical and axillary meristems, plants possess vascular meristems that generate xylem and phloem tissues during radial growth. These meristems are not located within structures ending in an acutely concave or convex surface. However, Lintilhac predicted a zone where tension and compression forces nullify in a disc-like geometry, similar to a transversal section of a shoot. This zone would correspond to a narrow ring, much like the ring of procambial and cambial cells that constitute the primary and secondary shoot and hypocotyl vascular meristems of *Arabidopsis* (Heywood, 1969; Lintilhac, 1974a,b) (Figure 4).

In the case of animals, like plant apical meristems, the SCN are located in tubular structures, close to a concave tip. Such is the case of the mammal intestinal crypts, hair follicles, *Drosophila* gonads, mammalian testis, etc. (Spradling et al., 2001; Barker et al., 2008). This suggests that SC could also be located at a mechanically isometric point in these animal systems. Moreover, there is now a critical amount of evidence showing that SC induction and maintenance is regulated by a variety of cues, including biomechanical ones: cell identity and activity has been shown to be regulated by mechanical forces acting on isolated animal cells, as well as by the interactions between cells and the ECM, interactions that are often mediated by integrins, focal adhesion proteins and the cytoskeleton (Ingber, 2006; and see excellent reviews in Guilak et al., 2009; Lee et al., 2011; Nava et al., 2012). Under this scenario, the stiffness, local geometry, and forces exerted on cells in their microenvironment emerge as crucial regulators of the position and function of SCs, both in plant and animal SCNs. There are, however, some animal SCs that are not located in notches or in the tip of tubular structures, as the mechanical isometry would predict, such as those found in the bone marrow or in the brain. It would be interesting to test whether the local cell-to-cell and cell-tissue interactions could create equivalent mechanical conditions in these contexts. Further experimental and theoretical explorations of this and related hypotheses also requires studies *in vivo*, as well as in other systems recently approached to study the emergence of the first multicellular body plans (Niklas et al., 2013; Niklas, 2014).

Besides mechanical isotropy, it has long been hypothesized that biochemical signals and fields generated by the cells around and inside the stem cell niches act as positional information determining the localization and identity of stem cells (e.g., Scheres, 2007). Among the biochemical processes that have been hypothesized to underlie cell-fate determination and patterning are the reaction-diffusion systems, which we briefly mention in the color patterning example as a complementary model. These systems are conformed by two chemicals, also known as morphogens, that react and diffuse at the same time, rendering heterogeneous and often periodic patterns of morphogen concentration. The hypothesis stating that morphogens or biochemical fields underlie stem-cell niche positioning has been tested both experimentally and theoretically, and seems to be complementary, rather than alternative, to that pointing to the role of mechanical force fields (see for example Newell et al., 2008; Barrio et al., 2013).

The terms *isotropy* and *isotropic point* are often used in the literature to refer to the mechanically special sites that appear to correspond to SCNs. However, the use of these terms may convey it has some limitations. For example, in the case of soft and non-homogeneous living materials with complex geometries, these special points can arise if forces going in opposite directions have the same magnitude, thus generating a null point. However, these forces need not be radially symmetric, and therefore the point would not be strictly isotropic. It will be important to consider this in the design of experimental test or further theoretical developments.

## Conclusions

Despite their divergent evolutionary history, plants and animals are largely formed by cells embedded in deformable fibrous media that, in close interaction with intracellular fibers, seem to constitute mechanically integral and self-sustained structures—tensegral structures. Given their unique organization, they can spontaneously reorganize in response to short- and long-range mechanical fields and, at the same time, transfer force to other fibers and contribute to the generation of these fields. In these tensegral matrices, mechanical stimuli generate and transduce position-dependent information during plant and animal development. The tensorial nature of mechanical forces provides spatial variation or directional information that cells perceive and that is not provided by the vector of a morphogen gradient (Wojtaszek, 2011) (Box 1).

Along this review we argue that the tensegrity principle might be a useful concept to integrate current data on the role of cell-to-cell and cell-tissue interactions during development, many of which remain largely unarticulated. This concept also allows to advance comparative studies in evolutionary developmental biology, as it provides a framework to contrast the key molecules and dynamics underlying the generation of tensegral structures and the emergence of position-dependent information during development of divergent multicellular organisms. We restate the hypothesis that the mechanically isometric points may be critical in the regulation of cell-shape and proliferation transitions in both plant and animal organs, particularly in stem cell niches.

Mathematical and computational models can be of great help to approach the study of the highly non-linear links between molecular, physico-chemical, cellular and tissular processes that affect each other during organismal development. Actually, some of these models suggest that the interaction between biochemical and mechanical processes add robustness to certain developmental processes, such as the direction of auxin fluxes and the establishment of phyllotactic patterns in plants (Newell et al., 2008). Similarly, cellular patterns in plants and animals emerge from the feedback dynamics of cell proliferation with chemical and mechanical fields, that are both important for the emergence of positional information. In an attempt to explore the role of coupled dynamics, Barrio et al. (2013) proposed a simple computational model for the *A. thaliana* root meristem. The model considers the relaxation of an elastic field, the transport and concentration gradient of auxins and the oscillations of the cell cycle regulators, and it seems to capture key aspects of the mechanisms underlying the emergent cell proliferation/elongation patterns along the root apical-basal axis. The authors assume an elastic field that can be characterized by point functions of stress, pressure or local mechanical forces, and that result from the symplastic nature of plant tissues formed by continuous cell-walls. Cell growth and proliferation in a physically constrained domain yield a lack of uniformity in the macroscopic mechanical field that, at the same time, results from, and constitutes a source of spatial information. Under such conditions, heterogeneous fields may elicit different responses of the signaling, genetic or metabolic networks in any biological system. In turn, the contrasting responses feedback to the physical, chemical and cell proliferation dynamics and patterns, and so on. This and other studies suggest that positional information is not external to the cellular dynamics, but rather results or emerges as a consequence of the feedbacks between the regulatory, signaling and metabolic networks, with the chemical and mechanical fields (Benítez et al., 2008; Alvarez-Buylla et al., 2009; Benítez and Alvarez-Buylla, 2010; Barrio et al., 2013; Bozorg et al., 2014).

Finally, despite the divergent evolutionary history, in both plants and animals cells are embedded in more or less deformable fibrous media that reorganizes in response to mechanical stimuli and, as a consequence, short- and long-range mechanical fields that generate and transduce position-dependent information emerge during plant and animal development. We argue that the tensegrity principle might be a useful concept to characterize and compare the structural and dynamic modules underlying the generation of this type of developmental patterns in both lineages of multicellular organisms.

### Conflict of interest statement

The authors declare that the research was conducted in the absence of any commercial or financial relationships that could be construed as a potential conflict of interest.
